# Association of vitamin D status with disease severity and outcome in Indian patients with IgA nephropathy

**DOI:** 10.1186/s12882-023-03061-0

**Published:** 2023-01-17

**Authors:** Naba Farooqui, Arunkumar Subbiah, Pradeep Chaturvedi, Hem Sati, Geetika Singh, Dipankar Bhowmik, Sanjay K. Agarwal, Soumita Bagchi

**Affiliations:** 1 All India Institute of Medical Sciences, New Delhi, India; 2 Department of Nephrology, All India Institute of Medical Sciences, New Delhi, India; 3 Department of Reproductive Biology, All India Institute of Medical Sciences, New Delhi, India; 4 Department of Biostatistics, All India Institute of Medical Sciences, New Delhi, India; 5 Department of Pathology, All India Institute of Medical Sciences, New Delhi, India

**Keywords:** 25 (OH) vitamin D, IgA nephropathy, Outcome

## Abstract

**Background:**

Vitamin D deficiency has been examined as a risk factor for severity and progression of kidney disease due to its immunomodulatory effects. There is paucity of data about its impact in IgA nephropathy (IgAN).

**Methods:**

In a retrospective cohort study, 25 (OH) vitamin D assay was performed in bio-banked baseline serum samples collected during kidney biopsy of 105 adult patients with primary IgAN diagnosed between 2015 and 2019. A level of < 10 ng/mL was defined as Vitamin D deficiency.

**Results:**

Mean age of patients was 34 ± 10.6 years, 69.5% were males. Mean baseline 25(OH) Vitamin D levels was 15.9 ± 11.9 ng/mL and 41(39%) patients had vitamin D deficiency. Serum albumin level was lower in vitamin D deficient patients compared to those who had higher vitamin D levels (3.7 ± 0.9 vs 4.1 ± 0.7 g/dl, *p* = 0.018)but there was no significant difference in baseline proteinuria and eGFR. Crescentic lesions were more frequent in vitamin D deficient group (19.5% vs 6.3%, *p* = 0.022). At median follow up of 21.5 months (6 – 56 months), there was no difference in remission (68.3% vs 65.6%, *p* = 0.777) and disease progression (12.5% vs 9.4%, *p* = 0.614) in those with and without Vitamin D deficiency respectively. On multivariate cox proportional hazard analysis, vitamin D deficiency was not a significant risk factor for renal survival (HR-1.79, 95% confidence interval:0.50–6.34, *p* = 0.368).

**Conclusion:**

There was no association between vitamin D deficiency and disease profile as well as renal outcome in Indian patients with IgAN.

## Introduction

IgA nephropathy (IgAN) is the most frequently diagnosed primary glomerular disease on renal biopsy in adults [1]. 20–30% of the patients progress to end stage kidney disease (ESKD) over a span of 10 to 20 years [2, 3]. However, we lack optimal non-invasive biomarkers to assess disease severity and prognosticate the outcome. Age, gender, hypertension, baseline renal function and proteinuria have been conventionally used as predictors of disease severity in clinical practice [4]. At present, therapeutic options comprise of supportive therapy with angiotensin converting enzyme inhibitor (ACEi) or an angiotensin receptor blocker (ARB) and blood pressure control followed by steroids and other immunosuppressive agents being used in those with progressive disease [5].

IgAN has been recognized to have an aggressive disease phenotype in Indians with 10-year survival reported to be around 35% [6–8].

Recent observations have highlighted the pleiotropic effects of Vitamin D. In patients with wide range of renal dysfunction, vitamin D deficiency was associated with vascular calcification, vascular endothelial function, cardiovascular events, and cardiovascular mortality [9–14]. Experimental data indicate that vitamin D analogues mediate a decrease in albuminuria and slow the progression of renal injury through activation of vitamin D receptor. Vitamin D insufficiency upregulates the renin-angiotensin system (RAS) and the NF-κB pathway, decreases the nitric oxide synthase transcription in vascular endothelial cells, increases inflammation and oxidative stress, and therefore may be a risk factor for progression of kidney disease. Vitamin D has been reported to play a role in preventing diabetic nephropathy and supplementation has shown to reduce proteinuria in these patients [15–17].

Vitamin D deficiency has been shown to corelate with severity of disease in IgAN [18]. Vitamin D supplementation with renin-angiotensin system blockade has been shown to reduce proteinuria in these patients [19–23]. Most of this information is from Chinese cohorts. Since there is considerable ethnic variability in the disease phenotype, role of Vitamin D on disease severity and outcome still remains uncertain in other populations. Vitamin D deficiency is widely prevalent in India [24, 25]. Being easily available, vitamin D assessment could serve as a useful additional factor to guide therapy if it affects disease outcome in IgAN patients.

## Methods

In a retrospective cohort study, we included adult patients (≥ 18 years) with biopsy proven primary IgAN diagnosed between 2015 and 2019 at a tertiary care referral institute in North India with a minimum follow-up of 6 months. We excluded patients who had (1) history of immunosuppression use in the previous 6 months before biopsy (2) secondary causes of IgAN like chronic liver disease, Henoch-Schonlein purpura (3) a second coexisting disease on kidney biopsy like diabetic nephropathy (4) inadequate/missing clinical records (5) inadequate kidney biopsy (6) follow up less than 6 months (7) no baseline serum sample available.

Serum 25-OH Vitamin D level was measured in serum samples drawn at the time of biopsy. These samples were obtained from a biorepository where we routinely store sera of patients collected at the time of kidney biopsy with their consent at -80^0^C until analysis with minimal freezing and thawing.

Serum 25(OH) vitamin D level was measured using the ARCHITECT™ assay which is a chemiluminescent microparticle immunoassay (CMIA). Vitamin D deficiency was defined as 25 (OH) vitamin D level < 10 ng/ml(severe deficiency). There is lack of consensus regarding the 25(OH) vitamin D level used to define vitamin D deficiency and the optimal target levels for different health outcomes [26, 27]. The prevalence of vitamin D deficiency is very high in India ranging from 70–100% in otherwise health populations using the standard definition of serum level of 25(OH)D < 20 ng/ml [24, 25]. In our study also 78(74.3%) patients had vitamin D deficiency based on this definition. So we decided to focus on patients with severe deficiency (25(OH)D < 10 ng/mL) to assess whether their outcome varied from the sub-group with higher levels.

Patients’ baseline demographic and clinical data and investigations including serum creatinine, albumin, presence of hematuria and proteinuria estimated by urine protein creatinine ratio (g/day), details of kidney biopsy and treatment given were retrieved from medical records. Estimated glomerular filtration rate (eGFR) was calculated by the Chronic Kidney Disease Epidemiology Collaboration (CKD-EPI) formula.

The primary outcome was renal disease progression defined as at least 50% decline in eGFR or progression to ESKD (eGFR < 10 ml/min/1.73m2 or requiring renal replacement therapy). Remission was defined as 24-h urine protein < 1 g/day with at least 50% decline from baseline and a stable renal function (≤ 25% decline in eGFR). The study was approved by the institute ethics committee, AIIMS, New Delhi and they provided a waiver of consent. The study was conducted according to the principles of declaration of Helsinki.

### Statistical analysis

Data were summarized as mean ± SD, frequency (%) or median (range). Chi-square test was used to compare the categorical variables while the continuous variables were compared between the groups using independent t-test or Wilcoxon rank-sum test. multivariate cox proportional hazards model was used to determine predictors of renal survival. Kaplan Meier event-free survival curves for patients with and without vitamin D deficiency were derived and compared. *P* values < 0.05 were considered significant. All analyses were performed using STATA 14.0 (StataCorp, College Station, TX).

## Results

One hundred and five patients with biopsy proven IgAN were enrolled in this study. The baseline profile of the study cohort is shown in Table 1. Their mean age was 34 ± 10.6 years and 69.5% were males. A significant proportion of our patients (65, 62%) were hypertensive. The baseline serum creatinine was 1.4 ± 0.5 mg/dl and eGFR was 72.2 ± 33.5 mL/min/1.73 m^2^. The mean serum albumin was 4.0 ± 0.8 g/dL with a proteinuria of 3.1 ± 2.5 g/day. The mean vitamin D level was 15.9 ± 11.9 ng/ml. 41 (39%) patients had vitamin D deficiency.

**Table 1:** Baseline characteristics of the study cohort

Study parameters	Total (*n* = 105)
Age at biopsy (yrs), mean ± SD	34 ± 10.6
Male (n/%)	73 (69.5)
Serum creatinine (mg/dL), mean ± SD	1.4 ± 0.5
eGFR (mL/min/1.73 m^2^), mean ± SD	72.2 ± 33.5
Proteinuria (g/day), mean ± SD	3.1 ± 2.5
Hypertension (n,%)	65 (62)
Serum albumin (g/dL) mean ± SD	4.0 ± 0.8
Serum uric acid (mg/dL) mean ± SD	6.8 ± 1.8
25 (OH) Vitamin D levels (ng/mL) mean ± SD, median with range	15.9 ± 11.9, 12(3.1,63.6)
Use of ACEi/ARBs^a^ (n/%)	92 (87.6)
Use of immunosuppressants (n/%)	60 (57.1)
Duration of follow-up (Months) (median, range)	21.5 (6 – 56)

^a^angiotensin converting enzyme inhibitors/angiotensin receptor blockers

The study cohort was divided into 2 groups based on vitamin D levels – vitamin D deficient group (25(OH)D < 10 ng/mL) and vitamin D replete group (25(OH)D ≥ 10 ng/mL). The various clinical and laboratory parameters were compared between these groups (Table 2). The vitamin D deficient group was younger (31.8 ± 9.3 vs 35.5 ± 11.2 years, *p* = 0.082), had more females [18 (43.9%) vs 14(21.9%), *p* = 0.017] and had higher eGFR though it was not statistically significant (78.0 ± 37.1 vs 68.4 ± 36.9 ml/min/1.73m2, *p* = 0.326) compared to the vitamin D replete group. Both groups had similar levels of proteinuria, but the vitamin D deficient group had lower serum albumin level (3.7 ± 0.9 vs 4.1 ± 0.7 g/dl, *p* = 0.018). Both groups had similar prevalence of hypertension. The use of ACEi/ARBs was also similar in both groups. Table 3 shows the Oxford MEST-C scores of the kidney biopsies of patients in the two groups. There was no difference in the proportion of M1, E1, S1 and T1/2 lesions between the two groups. Crescentic lesions were more frequent in the vitamin D deficient group (19.51% vs 6.25%, *p* = 0.022).

**Table 2:** Patient characteristics based on Vitamin D levels

Study parameters	25 (OH) Vitamin D		*p*-value
	< 10 ng/mL	≥ 10 ng/mL	
**Age**	31.8 ± 9.3	35.5 ± 11.2	0.082
**BMI (Kg/m2)**	24.8 ± 5.6	24.0 ± 3.1	0.335
**Female sex**	18 (43.9%)	14 (21.9%)	0.017
**Proteinuria (g/day)**	3.3 ± 2.0	3.0 ± 2.7	0.502
**Hypertension**	25 (60.1%)	40 (62.5%)	0.875
**eGFR** **(mL/min/1.73 m**^**2**^**), mean ± SD**	78.0 ± 37.1	68.4 ± 37.0	0.326
**eGFR < 60 mL/min/1.73 m2**	16 (39.0%)	31 (48.4%)	0.344
**Haematuria**	18 (28.1%)	29(70.7%)	0.887
**Serum albumin(g/dl), mean ± SD**	3.7 ± 0.9	4.1 ± 0.7	0.018
**Serum albumin < 3.5 g/dL**	14 (34.1%)	11 (17.2%)	0.047
**Use of ACE inhibitors**	38 (92.7%)	54 (84.4%)	0.242
**Use of Immunosuppressants**	25 (60.1%)	35 (54.7%)	0.525
**Renal disease progression (primary outcome)**	5(12.5%)	6 (9.4%)	0.614
**Disease Remission at follow-up**	28(68.3%)	42 (65.6%)	0.777

**Table 3:** Histologic characteristics based on vitamin D levels

	25(OH) Vitamin D < 10 ng/ml (*n* = 41)	25(OH) Vitamin D ≥ 10 ng/ml (*n* = 64)	P
M1(%)	29(70.7)	51(79.7)	0.293
E1(%)	1(2.4)	3(4.7)	1.00
S1(%)	29(70.7)	48(75.0)	0.629
T1/T2(%)	11(26.8) [T1-11, T2-0]	21(32.8) [T1-17, T2-4]	0.260
C1/C2(%)	8(19.5) [C1-8, C2-0]	4(6.3) [C1-3, C2-4]	0.022

**Fig. 1:**
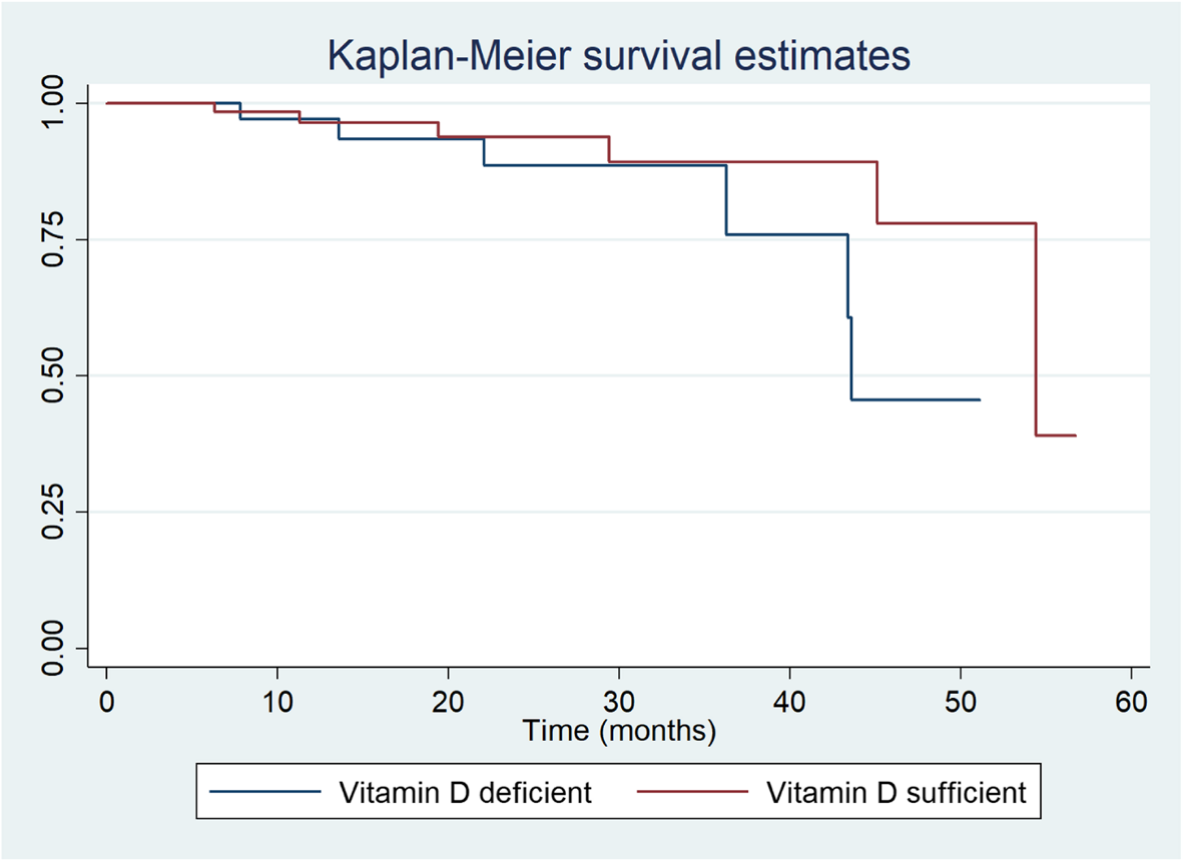
Kaplan–Meier renal survival estimates between patients with and without vitamin D deficiency

As shown in table 2, at follow-up, 5(12.5%) patients with vitamin D deficiency had progressed to the primary outcome as compared to 6(9.4%) patients with no vitamin D deficiency (*p* = 0.614). There was no difference in proportion of patients who achieved remission in those with and without vitamin D deficiency (68.3% vs 65.6%, *p* = 0.777). Univariate cox proportional hazard analysis of potential predictors of disease progression are shown in Table 4.Vitamin D deficiency at time of diagnosis was not a significant risk factor for renal disease progression (HR-1.79, 95% confidence interval:0.52–6.21, *p* = 0.357). Baseline vitamin D levels were also not predictive of disease progression on multivariate cox regression analysis(HR-1.79, 95% confidence interval:0.50–6.34, *p* = 0.368)There was no difference in time to adverse event i.e., loss of renal survival between patients with and without vitamin D deficiency as can be seen in the Kaplan Meier curve (Fig. 1).

**Table 4:** Factors at kidney biopsy predicting renal disease progression by univariate cox proportional hazards analysis

Baseline parameters	HR (95% CI)	*P*-value
Age	3.9 (0.75 –20.22)	0.105
Male sex	0.81 (0.23 – 2.82)	0.742
eGFR < 60 mL/min/1.73 m^2^	10.65 (1.36—83.23)	**0.02**
24 hour urinary protein ≥ 3.5 g/day	0.93 (0.27 – 3.24)	0.914
Hypertension	2.97 (0.64 – 13.80)	0.166
Serum albumin < 3.5 g/dL	1.78 (0.5 – 6.35)	0.377
Hematuria	0.27 (0.06 – 1.27)	0.097
No ACEi use	2.70 (0.74 – 9.86)	0.131
Absence of remission at follow-up	19.64 (2.5- 154.12)	**0.005**
IS** (not received)	0.64 (0.13–3.28)	0.596
Vitamin D deficiency	1.79 (0.52–6.21)	0.357

## Discussion

IgA nephropathy(IgAN) can become a challenging condition to manage as it is a smoldering disease. Considering the significant variability in clinical presentation and progression, many non-invasive biomarkers have been explored as prognostication tools with limited success. Vitamin D is known to have immune-modulatory effects. Due to its interaction with the renin angiotensin system and the NFkB pathways it may play a role in progression of kidney diseases with proteinuria. Baseline 25(OH) vitamin D level has been reported to be a predictor of disease progression and death in patients with stage 2–5 chronic kidney disease [9]. Vitamin D deficiency has also been co-related with proteinuria and worsening of kidney function in diabetics [15–17]. There is a paucity of information pertaining to the impact of vitamin D deficiency in IgAN and data mostly comes from Chinese studies. Li et al. found that lower baseline 25(OH) vitamin D levels not only had a significant correlation with poorer clinical outcomes and more severe renal pathological features but was also strongly associated with increased risk of renal progression [18]. Patients who had 25(OH) vitamin D level less than 15 ng/ml were categorized as being vitamin D deficient in this study. There were more females in the Vitamin D deficient group. The degree of proteinuria was similar in both groups though hypoalbuminemia was slightly more common in the deficient group. The mean eGFR was actually higher in those with vitamin D deficiency though it was not significant. The proportion of patients who achieved remission and had renal survival were similar in both groups. Li et al. [18] also showed a significant inverse association between vitamin D status and blood pressure which was not observed in our cohort. Tubulointerstitial chronicity(T1/T2) was more frequent in the vitamin D deficient subgroup(*p* = 0.008) in the Chinese study [18]. We did not observe this in our cohort. However, we found a y higher prevalence of crescentic lesions in our patients with Vitamin D deficiency.

Our study has certain limitations. It is retrospective, with a small cohort. It is not feasible to study the impact of baseline vitamin D deficiency on the outcome of patients unless bio-banked samples can be tested retrospectively. In a prospective to study it would be unethical to measure baseline 25(OH) vitamin D levels and not replenish those who are deficient thus making it difficult to interpret the impact on outcome. There is significant variability in vitamin D levels across geographies, ethnic groups and also different sampling seasons(winter vs summer months) leading to lack of agreement on cut-offs used to define deficiency and optimal target levels for different health outcomes. The prevalence of vitamin D deficiency is very high in India ranging from 70–100% in otherwise healthy populations using the standard definition of serum level of 25(OH)D < 20 ng/ml [24, 25]. In our study also 78(74.3%) patients had vitamin D deficiency based on this definition. So, we stratified patients based on the presence or absence of severe Vitamin D deficiency [26] to assess its prognostic significance. Li et al. [16] classified patients of IgAN with 25 (OH) vitamin D levels < 15 ng/ml as vitamin D deficient as in the Third National Health and Nutrition Examination Survey (NHANES III) cohort, this was associated with a higher risk for all-cause mortality in CKD patients [28]. The targets used to define vitamin D deficiency in different studies vary widely making comparisons difficult.

We also did not study the impact of vitamin D therapy on the outcome. In a randomized controlled trial of 50 patients, oral calcitriol with ACEi/ARB was found to reduce proteinuria in IgAN [20]. Whether this is due to the effect of vitamin D deficiency in the pathogenesis of IgAN or due to its interaction with the renin-angiotensin system needs to be determined. Our study suggests that vitamin D deficiency per se may not impact disease outcome in IgAN patients. Unnecessary treatment may lead to adverse effects due to vitamin D intoxication. We need to study larger cohorts with longer follow-up to ascertain its role in the disease pathway of IgAN.

## Data Availability

All data required for this study and the relevant analysis are included in the manuscript. Any additional data required may be made available from the corresponding author on reasonable request.
